# Janus-faced role of SIRT1 in tumorigenesis

**DOI:** 10.1111/j.1749-6632.2012.06762.x

**Published:** 2012-10-10

**Authors:** Na-Young Song, Young-Joon Surh

**Affiliations:** 1 Tumor Microenvironment Global Core Research Center, College of PharmacySeoul, South Korea; 2 Department of Molecular Medicine and Biopharmaceutical Sciences, Graduate School of Convergence Sciences and TechnologySeoul, South Korea; 3 Cancer Research Institute, Seoul National UniversitySeoul, South Korea

**Keywords:** SIRT1, cancer, subcellular localization

## Abstract

Silent mating type information regulation 1 (Sirtuin 1; SIRT1) has been reported to regulate various physiological events, such as aging and metabolism, via deacetylation of histone and nonhistone proteins. Notably, cumulative evidence supports the notion that SIRT1 has a Janus-faced role in tumorigenesis. SIRT1 contributes to anti-inflammation, genomic stability, and cancer cell death, and hence it has tumor-suppressor properties. On the other hand, SIRT1 can stimulate oncogenic signaling pathways and can create a tumor microenvironment favorable to growth and survival of cancer cells. Such dual functions of SIRT1 may be determined, at least in part, by its subcellular localization. Interestingly, SIRT1 displays differential localization in normal cells and cancer cells, which in turn may affect the substrate specificity for its deacetylase activity.

## Introduction

Sirtuins are the mammalian orthologues of yeast silent information regulator 2 (SIR2) that have been found to extend yeast life span.^1^ The sirtuin protein family consists of seven isoforms, SIRT1 to SIRT7, which have specific subcellular localizations and activities.^2^ SIRT1, the most well-characterized member of the sirtuin family, has been known as a longevity protein in the mammals, particularly related to life-span extension induced by caloric restriction.^3^,^4^ Resveratrol, a phytoalexin found in grapes, mimics the effect of caloric restriction, which has been speculated to be mediated through activation of SIRT1.^5^–^7^ A recent study has revealed that SIRT1 is involved in the beneficial effects of resveratrol on mitochondrial function.^8^ However, it is still unclear whether resveratrol activates SIRT1 directly or indirectly.

SIRT1 belongs to the family of NAD^+^-dependent class III histone deacetylases.^9^,^10^ The deacetylation targets of SIRT1 are not limited to histones but are expanded to diverse proteins, including the tumor suppressor p53.^11^ Lysine acetylation and deacetylation have been recognized as crucial events for regulation of activity, stability, and subcellular localization of proteins.^12^ Thus, SIRT1 can modulate various cellular signaling pathways through alteration of the acetylation status of target proteins.

Deacetylation activity of SIRT1 can be modulated by multiple regulators. Two putative regulators are active regulator of SIRT1 (AROS) and deleted in breast cancer 1 (DBC1), which are positive and negative regulators of SIRT1, respectively. While the nuclear protein AROS directly binds to SIRT1, leading to enhanced deacetylase activity of the latter protein,^13^ DBC1 interacts with the catalytic domain of SIRT1 and negatively regulates SIRT1-dependent deacetylation.^14^ However, the exact roles of AROS and DBC1 as SIRT1 modulators need to be confirmed.

Furthermore, posttranslational modifications of SIRT1 affect its deacetylase activity. Sumoylation of SIRT1 at residue Lys 734 facilitates its catalytic activity, which is diminished by SENP1 desumoylase.^15^ Phosphorylation is also important for SIRT1 activity. Cyclin-dependent kinase 1 and c-Jun N-terminal kinase 1 have been reported to phosphorylate SIRT1, positively modulating its activity.^16^,^17^ These complex factors coordinately regulate SIRT1 activity and subsequent cellular events.

Functioning as a protein deacetylase, SIRT1 has a broad spectrum of substrates. The tumor suppressor p53 is one of the best-defined target proteins of the SIRT1 deacetylase.^11^ SIRT1 deacetylates and inactivates p53, thereby exerting an antiapoptotic effect.^18^ Moreover, SIRT1 can be involved in the DNA repair process through deacetylation of Ku70.^19^ SIRT1 also deacetylates and activates liver X receptor proteins, facilitating cholesterol efflux from the cell.^20^ Peroxisome proliferator-activated receptor γ coactivator 1α (PGC-1α) can modulate metabolic pathways by directly interacting with several transcription factors, such as peroxisome proliferator-activated receptor γ. SIRT1-dependent deacetylation of PGC-1α enhances its ability to cooperate with transcription factors, which, in turn, induces expression of genes involved in fatty acid oxidation and gluconeogenesis.^21^,^22^

**Figure 1:**
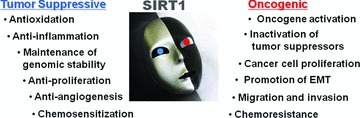
The Janus face of SIRT1 in tumorigenesis. SIRT1 can inhibit inflammation and multistage carcinogenesis, acting as a tumor suppressor. Paradoxically, SIRT1 also accelerates tumorigenesis via multiple mechanisms, such as inactivation of tumor suppressors, activation of oncoproteins, and development of a microenvironment favorable for tumor survival.

Although the majority of investigations concern anticarcinogenic as well as antiaging effects of SIRT1, recent studies have revealed that the protein is implicated in carcinogenesis (Fig. 1). However, the exact role of SIRT1 in carcinogenesis is still controversial. This review highlights the Janus-faced role of SIRT1 in multistage carcinogenesis.

## SIRT1 as a putative tumor suppressor

### SIRT1 inhibits aberrantly amplified proinflammatory signaling during promotion and progression of carcinogenesis

It is well known that chronic inflammation is associated with carcinogenesis, especially in the promotion and progression stages.^23^ SIRT1 can inhibit inflammation provoked by several proinflammatory cytokines, such as tumor necrosis factor α (TNF-α), lipopolysaccharide (LPS), and interleukins (ILs). The natural SIRT1 activator resveratrol has been shown to attenuate TNF-α–induced inflammation in mouse embryonic fibroblasts (MEFs) through upregulation of SIRT1.^24^ Resveratrol also ameliorates inflammation provoked during acute and restorative phases in murine liver tissues, while it failed to overcome LPS challenge in the liver of SIRT1 knockout mice.^25^ Moreover, SIRT1-knockdown macrophages exhibited increased inflammatory responses.^26^ Hepatic steatosis and inflammation were evident in SIRT1 knockout mice, lending further support to the anti-inflammatory role of SIRT1.^27^,^28^ SIRT1 expression was also found to be reduced in the lungs of patients with chronic obstructive pulmonary disease that is closely related to chronic inflammation.^29^

The anti-inflammatory effect of SIRT1 might be achieved by inhibition of several transcription factors related to inflammation. Nuclear factor κB (NF-κB) is a key transcription factor responsible for regulation of immune responses. SIRT1 directly interacts with the RelA/p65 subunit of NF-κB, leading to deacetylation and subsequent inactivation of NF-κB.^30^ However, siRNA knockdown of SIRT1 augmented acetylation of RelA/p65, as well as release of IL-8, in a monocyte–macrophage cell line.^29^ In the dextran sodium sulfate-induced murine colitis model, resveratrol upregulated SIRT1 expression and abrogated NF-κB activation, thus attenuating intestinal inflammation.^31^ In addition to NF-κB, SIRT1 inhibits other transcription factors, including those that orchestrate proinflammatory responses. Signal transducer and activator of transcription 3 (STAT3) is phosphorylated and activated in response to various proinflammatory cytokines, consequently promoting inflammation-associated carcinogenesis.^32^ STAT3 has been demonstrated as a binding partner as well as a substrate of SIRT1.^33^ Indeed, overexpression of SIRT1 inhibited acetylation, phosphorylation, and transactivation of STAT3;^33^ in contrast, silencing of SIRT1 potentiated IL-22–driven acetylation and phosphorylation of STAT3. These results are indicative of the existence of interplay between SIRT1 and STAT3.^34^ Similarly, activator protein 1 (AP-1) is inactivated through direct interaction with SIRT1, though it is unclear whether SIRT1-depdendent deacetylation is involved in this process.^35^ Based on the findings, one can conclude that SIRT1 inhibits proinflammatory signaling that is often inappropriately activated in transformed and cancerous cells; thus, it can be anticipated that SIRT1 inhibits inflammation-associated carcinogenesis.

### SIRT1 exerts anticarcinogenic effects through multiple mechanisms

Besides inhibiting abnormally activated proinflammatory signaling and subsequently preventing inflammation-associated cancer, SIRT1 participates in suppression of multistage carcinogenesis via other mechanisms. For example, SIRT1 can counteract various genotoxic insults, including oxidative DNA damage, thereby blocking initiation of carcinogenesis. SIRT1 deacetylates and inhibits proapoptotic p53 and poly (ADP-ribose) polymerase 1 under stressful conditions, conferring adaptive cell survival.^18^,^36^ SIRT1 is also required for DNA repair processes, both nuclear excision repair and double-strand break repair, to maintain genomic stability.^37^,^38^ Furthermore, SIRT1-overexpressing MEFs showed longer telomeres, whereas telomere shortening was observed in SIRT1-deficient MEFs.^39^

Inappropriate overexpression of the cellular oncogene, such as c-Myc, is evident in some human malignancies; c-Myc binds to the SIRT1 promoter and induces SIRT1 expression. However, SIRT1 interacts with and deacetylates c-Myc, resulting in decreased c-Myc stability. 40 As a consequence, the transforming activity of c-Myc is compromised. The downregulation of c-Myc–mediated cellular transformation via a c-Myc–SIRT1 negative feedback loop supports a role of SIRT1 in tumor suppression.^40^ SIRT1 can hamper cancer cell proliferation as well. Ectopic overexpression of SIRT1 suppressed G418-resistant colony formation of human colon cancer HCT-116 cells.^41^ Resveratrol inhibited growth of several human cancer cell lines, which was possibly mediated by SIRT1-depedent relocalization of Werner syndrome protein (WRN) from the nucleolus to the nucleoplasm;^42^ in contrast, the SIRT1 inhibitor EX-527 stimulated DNA replication and proliferation of cancer cells.^41^ In addition to growth arrest, resveratrol triggered apoptosis of BRCA1 mutant tumor cells through SIRT1-dependent inhibition of survivin.^43^ SIRT1 also promoted autophagy in prostate cancer, based on the observation that overexpression of SIRT1 resulted in accumulation of autophagy-related proteins, while the SIRT1 antagonist sirtinol repressed autophagy.^44^

Moreover, SIRT1 can deacetylate and inactivate hypoxia-inducible factor 1α (HIF-1α), a transcription factor that can rescue solid tumors from hypoxic burden through facilitation of angiogenesis and metastasis.^45^ SIRT1 inhibits HIF-1α–mediated expression of oncoproteins. The tissue levels of vascular endothelial growth factor, a key stimulator of angiogenesis, were markedly reduced in xenograft tumor of SIRT1-overexpressing human sarcoma cells.^45^ SIRT1 negatively regulated matrix metalloproteinase-9, an enzyme responsible for cancer cell invasion and migration.^46^ Brouguignon *et al.* have shown that reveratrol acts as a chemosensitizer by repressing multidrug resistance protein 1 (MDR1) in a SIRT1-dependent manner.^47^

The anticarcinogenic effects of SIRT1 have been demonstrated in numerous *in vivo* studies. When nude mice were implanted with SIRT1-overexpressing cells, tumor growth was suppressed.^40^,^45^ In contrast, significant enlargement of xenograft tumor was observed in nude mice that had received SIRT1-knockdown colon cancer cells.^41^ Wang *et al*. have demonstrated that SIRT1^+/−^ p53^+/−^ double mutant mice spontaneously generate tumors at five months of age and show a higher incidence of tumors in multiple organs, compared with wild-type, SIRT1^+/−^ and p53^+/−^ mice.^48^ Moreover, SIRT1-overexpressing *APC*^min/+^ mice developed fewer intestinal tumors compared with SIRT1 wild-type mice, further supporting the anticarcinogenic function of SIRT1.^49^

## Oncogenic functions of SIRT1

### SIRT1 is overexpressed in some tumors

On the premise that SIRT1 acts as a tumor suppressor, SIRT1 could be downregulated in many tumors. In support of this speculation, SIRT1 expression was found to be reduced in human skin tumors.^37^ Paradoxically, however, elevated expression of SIRT1 has been observed in various types of human malignancies. The immunostaining of SIRT1 has revealed that both the proportion of positive cells and staining intensity are significantly increased in human prostate cancer specimens.^50^ SIRT1 was also highly expressed in other types of human cancer tissues, such as ovary, liver, breast, stomach, and pancreas.^51^–^55^ In case of human colorectal cancer, SIRT1 overexpression was detected as well.^56^ However, other investigations have revealed pronounced SIRT1 expression in both normal colon and tumor tissues, even though expression is substantially reduced in some higher grade colon tumors.^41^,^57^

In spite of the conflicting observations, SIRT1 overexpression in tumors has some clinical implications. Thus, upregulation of SIRT1 in tumors was found to be associated with unsatisfactory therapeutic outcomes in some cancer patients. Kaplan–Meier analyses of survival in cancer patients with or without SIRT1 overexpression revealed that those in the SIRT1-positive group exhibited poor prognosis outcome and shorter overall survival.^54^,^58^–^60^ In addition to survival time, SIRT1 overexpression was correlated with the higher tumor stage and the presence of lymph node metastasis in gastric and pancreatic cancer patients.^55^,^60^ SIRT1-positive tumors showed higher expression of Ki-67, a cell proliferation marker, which may account for the poor prognosis in SIRT1-positive cancer patients.^54^ Interestingly, DBC1, a negative regulator of SIRT1 activity, was coordinately overexpressed together with SIRT1 in gastric cancer patients,^60^ which is considered compensatory expression for abnormal upregulation of SIRT1.

### SIRT1 deregulates both tumor suppressors and oncoproteins

Overexpression of SIRT1 may aggravate tumorigenesis through abnormal modulation of certain proteins, particularly tumor-suppressor proteins and (proto)oncogenes. The tumor suppressor p53 is the most representative substrate of SIRT1 deacetylase. In response to genotoxic insults, p53 upregulated a potential tumor suppressor microRNA 34a (miR-34a), thus promoting apoptosis.^61^ However, SIRT1 might facilitate deacetylation and inactivation of p53 in cancerous cells, leading to repression of miR-34a.^62^ Hypermethylated in cancer 1 (HIC1) is also a tumor suppressor that cooperates with p53 to induce apoptosis in response to DNA damage.^63^ Primary tumors derived from HIC1^+/−^ mice exhibited SIRT1 overexpression.^63^ Moreover, SIRT1 has been reported to deacetylate retinoblastoma (Rb) protein and phosphatase and tensin homologue deleted in chromosome 10 (PTEN), repressing their tumor suppressive activity.^64^,^65^

In addition to inactivation of tumor suppressors, SIRT1 overexpression is associated with deregulation of protooncogenes. Myc genes are well-known prototypic oncogenes.^66^ SIRT1-dependent deacetylation of c-Myc can enhance its stability, association with c-Max, and transcriptional activity.^67^,^68^ Furthermore, SIRT1 also deacetylates N-Myc, resulting in stabilization of this oncoprotein and cell proliferation.^69^ In turn, these Myc proteins can upregulate SIRT1 expression, consequently forming positive feedback loops between Myc proteins and SIRT1.^67^,^69^ These findings are opposite to the previously reported destabilization of c-Myc by SIRT1 as a consequence of deacetylation of c-Myc through direct interaction between two entities.^40^

Moreover, SIRT1 is a downstream of oncogenic BCR-ABL tyrosine kinase. The SIRT1 silencing suppressed BCR-ABL-mediated transformation of bone marrow cells and development of a chronic myelogenous leukemia (CML)-like myeloproliferative disease.^70^ Recently, it has been reported that SIRT1 positively regulates membrane localization and oncogenic activation of Akt via deacetylation.^71^ The Ras oncoproteins appear to play a role in SIRT1-associated tumorigenesis as well. The SIRT1 inhibitor sirtinol suppressed Ras activation, but it remains to be elucidated which protein is a *bona fide* target of SIRT1 for its oncogenic functions.^72^ Collectively, SIRT1 can be involved in carcinogenesis through deregulation of tumor suppressors and prototypic oncoproteins.

### SIRT1 confers survival advantages to cancer cells

SIRT1 promotes cancer cell proliferation and survival. SIRT1-overexpressing hepatocellular carcinoma Hep1 cells showed enhanced proliferation, whereas there was no change in Hep1 cells transfected with a deacetylase-defective SIRT1 H363Y mutant construct.^52^ In contrast, SIRT1 inhibition abrogated colony formation in human breast cancer MCF-7, lung cancer H1299, and CML progenitor cells.^72^,^73^ Likewise, SIRT1 inhibitors have been reported to trigger cell death in various types of human cancer cell lines.^72^,^74^–^76^ Suppression of cancer cell proliferation as a consequence of SIRT1 downregulation might be attributable to telomere dysfunction and increased acetylation and subsequent activation of p53.^73^,^77^ The stimulation of cell proliferation by SIRT1 seems to be cancer specific. Ford *et al.* have shown that siRNA knockdown of SIRT1 leads to enhanced apoptosis of various cancer cell lines, whereas it fails to affect apoptosis or growth arrest in normal human epithelial cell lines and normal primary diploid fibroblasts.^78^

Epithelial to mesenchymal transition (EMT) is recognized as a plausible mechanism of tumor progression and the invasion-metastasis cascade.^79^ Recently, SIRT1 has emerged as a regulator of EMT-like transformation in tumors; for example, it has been demonstrated that SIRT1 is upregulated in human mammary epithelial cells during the EMT induced by tumor growth factor β.^80^ Conversely, SIRT1 silencing reduced expression of ZEB1, an EMT-inducing transcription factor, while restoring E-cadherin expression that is generally downregulated during the EMT process.^80^,^81^ SIRT1 thus plays a crucial role in EMT-associated signal transduction. SIRT1 silencing has also restored cell–cell adhesion, while reducing the invasiveness of cancer cells.^55^,^81^ Moreover, ectopic overexpression of SIRT1 enhanced migration of SIRT1-null MEFs, suggesting that SIRT1 directly promotes cell migration.^51^

SIRT1-dependent deacetylation of cell motility proteins is thought to be a feasible mechanism underlying SIRT1-promoted invasion and migration. Cortactin, an F-actin binding protein, promotes cell migration once it is acetylated.^51^ SIRT1 physically interacts with and deacetylates cortactin, resulting in enhanced cell migration.^51^ Furthermore, Dishevelled (Dvl) proteins involved in Wnt signaling also take part in SIRT1-dependent cell migration. Dvl proteins form a complex with SIRT1 and then undergo deacetylation and subsequent stabilization.^82^ SIRT1-dependent positive regulation of Dvl proteins seems to be crucial for Wnt-mediated cell migration. Genetic or pharmacologic inhibition of SIRT1 attenuated expression of Wnt downstream proteins and concomitantly Wnt-induced cell migration.^82^

Furthermore, SIRT1 contributes to acquisition of chemoresistance in several types of tumors. Various drug-resistant cancer cell lines have exhibited overexpression of SIRT1, suggesting that SIRT1 may take part in chemoresistance.^83^,^84^ In line with this notion, SIRT1 gain-of-function activity is associated with upregulated expression of MDR1, a major drug resistance molecule, in HEK293 cells.^83^ Conversely, inhibition of SIRT1 by use of pharmacological inhibitors or siRNA knockdown reduced MDR1 expression in cancer cells, thus enhancing chemosensitivity.^74^,^83^,^85^ The *in vivo* xenograft assay using doxorubicin-resistance MCF-7 cells also showed augmentation of doxorubicin responsiveness by a SIRT1 inhibitor amurensin G.^85^ In the case of CML, SIRT1 deacetylates DNA repair proteins, such as Nijmegen breakage syndrome protein 1 and Ku70, resulting in acquisition of BCR–ABL mutations and subsequent drug resistance.^86^ Moreover, SIRT1 renders cancer cells resistant to radiation-induced apoptosis.^87^ Based on these observations, it seems likely that SIRT1 overexpression confers survival advantages to cancerous or transformed cells and accelerates tumorigenesis.

## Subcellular localization may account for differential roles of SIRT1 in normal versus cancer cells

**Figure 2:**
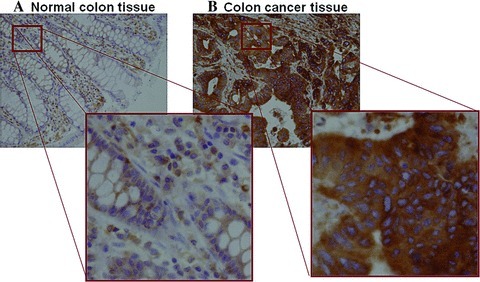
Differential subcellular localization of SIRT1 in a human colon tumor specimen and normal colonic tissues. SIRT1 is highly expressed in human colon tumor tissues (B) compared with normal counterparts (A). Notably, SIRT1 showed cytoplasmic localization in colon tumors (B), whereas the adjacent normal tissues displayed rather nuclear-concentrated expression (A).

As mentioned earlier, SIRT1 has dual effects on carcinogenesis (Fig. 1). SIRT1 has been reported to inhibit inflammation, transformation, tumor promotion, and progression. However, SIRT1 also exerts opposite effects, acting as a tumor promoter. Such a double-edged sword nature of SIRT1 might be potentially determined by its subcellular localization. SIRT1 has been identified as a nuclear protein at first.^10^ However, it has been demonstrated that SIRT1 contains at least two nuclear localization signals (NLSs) and two nuclear export signals (NESs) and hence undergoes nucleocytoplasmic shuttling.^88^ This implies that SIRT1 can be located in cytosol as well as nucleus. SIRT1 was found to be normally present in the cytoplasm in murine pancreatic islet cells and human embryonic kidney cells.^89^,^90^ Notably, SIRT1 was overexpressed predominantly in the cytosol of certain cancer cells, while normal epithelial cells showed nuclear localization.^56^,^91^ This phenomenon has been also observed in human colon and ovarian cancer specimens.^51^,^56^Figure 2 illustrates differential expression of SIRT1 in human colon tumor and surrounding tissues.

**Figure 3:**
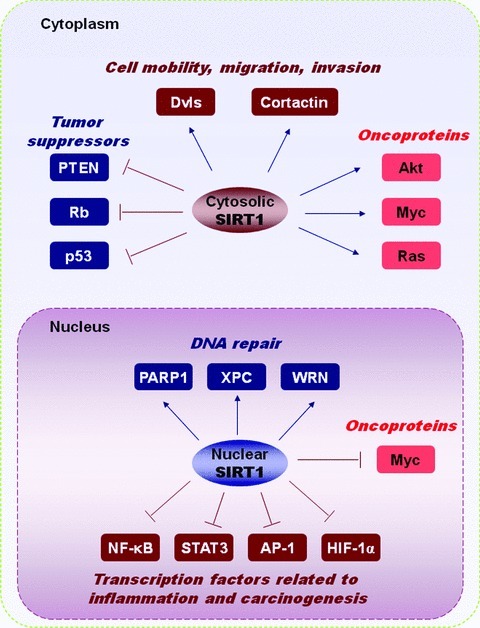
Some represantive intracellular proteins modulated by SIRT1. Nuclear and cytoplasmic SIRT1 may differentially target proteins as deacetylation substrates. Thus, SIRT1 may exert specific functions, depending on its subcellular localization.

The differential subcellular localization of SIRT1 may affect the substrate specificity in normal versus cancer cells. As illustrated in Figure 3, substrates of the SIRT1 deacetylase can be classified into two groups, cytoplasm- and nucleus-predominant, according to their subcellular localization. In the normal cells, SIRT1 seems to be present mainly in the nucleus, predominantly targeting nuclear proteins. Nuclear SIRT1 can deacetylate and inactivate transcription factors, including NF-κB, STAT3, HIF-1α, and AP-1, exerting anti-inflammatory and anticarcinogenic effects.^30^,^33^,^35^ Moreover, nuclear SIRT1 is also involved in maintenance of genomic stability through deacetylation of DNA repair proteins, such as PARP1, XPC, and WRN.^36^,^37^,^42^ SIRT1 has been reported to form both negative and positive feedback loops with c-Myc, which appear to be related to its anti- and procarcinogenic actions, respectively.^40^,^67^,^68^ This might be attributed to the different localization of both SIRT1 and c-Myc in normal cells and in cancer cells. Similar to SIRT1, Myc oncoproteins normally reside in the nucleus, yet cancer cells show overexpression of Myc proteins predominantly in the cytoplasm.^66^,^92^ In the nucleus of normal cells, SIRT1 deacetylates and destabilizes c-Myc, suppressing c-Myc-driven tumorigenesis.^40^ However, SIRT1 shows cytoplasmic localization in cancer cells, targeting cytosolic proteins as its preferred deacetylation substrates.^56^,^91^ Thus, SIRT1 can deacetylate and stabilize Myc proteins, promoting tumorigenesis.^67^ Cytoplasmic SIRT1 may also deacetylate and activate the Akt oncoprotein.^71^ Moreover, SIRT1 can deacetylate cytoplasmic proteins involved in locomotion, including cortactin and Dvl proteins, leading to enhanced cell mobility.^51^,^82^ Interestingly, p53, a well-known target of SIRT1, accumulates in the cytoplasm of various cancer cells, functioning as a proapoptotic protein; and SIRT1 might inhibit p53-dependent apoptosis in the cytoplasm of cancer cells.^93^,^94^

## Concluding remarks

SIRT1, a NAD^+^-dependent histone/protein deacetylase, has been emerging as a crucial regulator of assorted physiological events through deacetylation of various proteins related to apoptosis, DNA repair, and metabolism.^10^,^95^ SIRT1 is also involved in tumorigenesis; but it is still under much debate whether SIRT1 stimulates or suppresses carcinogenic processes (see Fig. 1). SIRT1 inhibits inflammation and activities of transcription factors that exacerbate carcinogenesis.^30^,^33^,^35^,^49^ Moreover, SIRT1 contributes to preservation of genomic stability.^38^,^39^ Thus, SIRT1 takes part in prevention, retardation, and suppression of carcinogenesis. SIRT1 is supposed to be underexpressed in tumors if it is indeed a tumor suppressor. Contrary to this supposition, substantial proportions of human cancer specimens have shown overexpression of SIRT1.^51^–^55^ SIRT1 aggravates inflammation, inactivates tumor suppressors, and, concomitantly, activates protooncogenes.^63^,^65^,^71^,^96^ In addition, SIRT1 promotes cancer cell proliferation, invasion, migration, and chemoresistance, conferring survival advantages to cancer cells.^51^,^52^,^80^

The subcellular localization of SIRT1 might be responsible, at least in part, for determination of its dual roles in tumorigenesis. However, there should be factors other than subcellular localization that modulate SIRT1 functions. For instance, the complex regulators of the SIRT1 activity, such as AROS and DBC1, should be considered. Moreover, SIRT1 deacetylation of c-Myc was found to modulate the stability of this oncoprotein in both positive and negative manners.^40^,^67^,^68^ In this case, the different subcellular localization of SIRT1 was insufficient to assign to it reciprocal regulation of c-Myc protein stability. Although the subcellular locailization of SIRT1 is not a sole determinant of functions of this deacetylase in tumorigenesis, it is evident that the nuclear and cytoplasmic SIRT1 might exert distinct functions. Cytoplasmic SIRT1 promoted neurite outgrowth in PC12 cells, which was inhibited by the nuclear SIRT1.^97^ Furthermore, a SIRT1 NLS mutant unable to enter the nucleus failed to suppress colony formation, whereas SIRT1-overexpressing cells showed a strong inhibitory effect.^41^

In conclusion, SIRT1 might modulate tumorigenesis in both positive and negative manners, partially depending on its subcellular localization. However, further investigation is required to fully clarify whether subcellular localization of SIRT1 is indeed a fate-determinant of its oncogenic versus tumor-suppressing functions. In particular, it is important to determine whether the SIRT1 NLS and/or NES contain point mutation(s) in human cancer tissues. In addition, SIRT1 mutations within the NLS and/or NES domain(s) would be useful tools for better elucidating the specific functions of nuclear and cytoplasmic SIRT1.
